# The G-protein-coupled bile acid receptor Gpbar1 (TGR5) protects against renal inflammation and renal cancer cell proliferation and migration through antagonizing NF-κB and STAT3 signaling pathways

**DOI:** 10.18632/oncotarget.17533

**Published:** 2017-04-29

**Authors:** Jia Su, Qiqi Zhang, Hui Qi, Linlin Wu, Yuanqiang Li, Donna Yu, Wendong Huang, Wei-Dong Chen, Yan-Dong Wang

**Affiliations:** ^1^ State Key Laboratory of Chemical Resource Engineering, College of Life Science and Technology, Beijing University of Chemical Technology, Beijing, P.R. China; ^2^ Key Laboratory of Receptors-Mediated Gene Regulation and Drug Discovery, School of Medicine, Henan University, Kaifeng, Henan, P.R. China; ^3^ Key Laboratory of Molecular Pathology, School of Basic Medical Science, Inner Mongolia Medical University, Hohhot, Inner Mongolia, P.R. China; ^4^ Department of Diabetes and Metabolic Diseases Research, Beckman Research Institute, City of Hope National Medical Center, Duarte, California, USA

**Keywords:** Gpbar1, TGR5, renal inflammation, STAT3, NF-κB

## Abstract

Gpbar1 (TGR5), a G-protein-coupled bile acid membrane receptor, is well known for its roles in regulation of glucose metabolism and energy homeostasis. In the current work, we found that TGR5 activation by its ligand suppressed lipopolysaccharide (LPS)-induced proinflammatory gene expression in wild-type (WT) but not TGR5^−/−^ mouse kidney. Furthermore, we found that TGR5 is a suppressor of kidney cancer cell proliferation and migration. We show that TGR5 activation antagonized NF-κB and STAT3 signaling pathways through suppressing the phosphorylation of IκBα, the translocation of p65 and the phosphorylation of STAT3. TGR5 overexpression with ligand treatment inhibited gene expression mediated by NF-κB and STAT3. These results suggest that TGR5 antagonizes kidney inflammation and kidney cancer cell proliferation and migration at least in part by inhibiting NF-κB and STAT3 signaling. These findings identify TGR5 may serve as an attractive therapeutic tool for human renal inflammation related diseases and cancer.

## INTRODUCTION

Inflammation is recognized as one of important features of chronic kidney disease (CKD) [1]. Chronic inflammation is the major mediators of progression of renal disease and the associated cardiovascular disease, anemia, cachexia, and many other complications in CKD patients [2]. There is still no effective treatment for CKD, which resulted in 956,000 deaths in 2013 [3]. A better understanding of the mechanisms underlying the development of CKD and discovery of novel approaches for treatment of CKD are urgent.

NF-κB has been regarded as a key regulator of inflammation because activation of NF-κB can be detected in inflammation-associated diseases including cancer [4–6]. NF-κB is rapidly activated by some cytokines. Recent studies have shown that NF-κB is an important regulator of CKD [2, 7]. The classic form of NF-κB consists of a heterodimer of p65 (RelA) and p50. Kuhad et al. reported that the renal function decrease is associated with increased active p65 subunit of NF-κB [8]. Tumur et al. reported that the upregulation of canonical (RelA/p50) isoform of NF-κB by indoxyl sulfate exacerbated inflammatory status in renal disease [9]. Rangan et al. in their report indicated the importance of upregulating of NF-κB and its subunit p65 in mediating chronic inflammation in CKD [10]. These reports suggest NF-κB signaling plays the key functions in progression of CKD.

Signal transducer and activator of transcription 3 (STAT3) is also a key player of inflammation and cancer [11–13]. Activation of STAT3 after stimulation by various growth factors and cytokines requires transient phosphorylation of cytoplasmic monomers [15]. STAT3 activation is tightly controlled in normal conditions. Conversely, extensive and chronic STAT3 activation is frequently detected in many human inflammation-associated diseases including CKD [16]. Bienaime et al. reported that Stat3 signaling is important in progression of CKD [17]. Therefore, finding new targets that suppress STAT3 is crucial for developing novel strategies to improve therapeutic potential for CKD.

TGR5 belongs to the G-protein-coupled receptor (GPCR) family. It is a regulator of bile acid and energy homeostasis, as well as glucose metabolism [18, 19]. We previously shown that TGR5 suppressed gastric and liver inflammation via inhibiting NF-κB pathway [6, 20]. In the current work, we show that activation of TGR5 by its ligand suppressed proinflammatory gene expression induced by LPS in wild-type (WT) kidney. We found that TGR5 inhibited STAT3 phosphorylation and IκBα phosphorylation in STAT3 and NF-κB pathways of kidney cells, respectively. These results suggest the ligands of TGR5 may be used for treatment of human kidney diseases through inhibiting STAT3 and NF-κB signaling.

## RESULTS

### TGR5 activation suppresses expression of proinflammatory genes in mouse kidney

**Figure 1:**
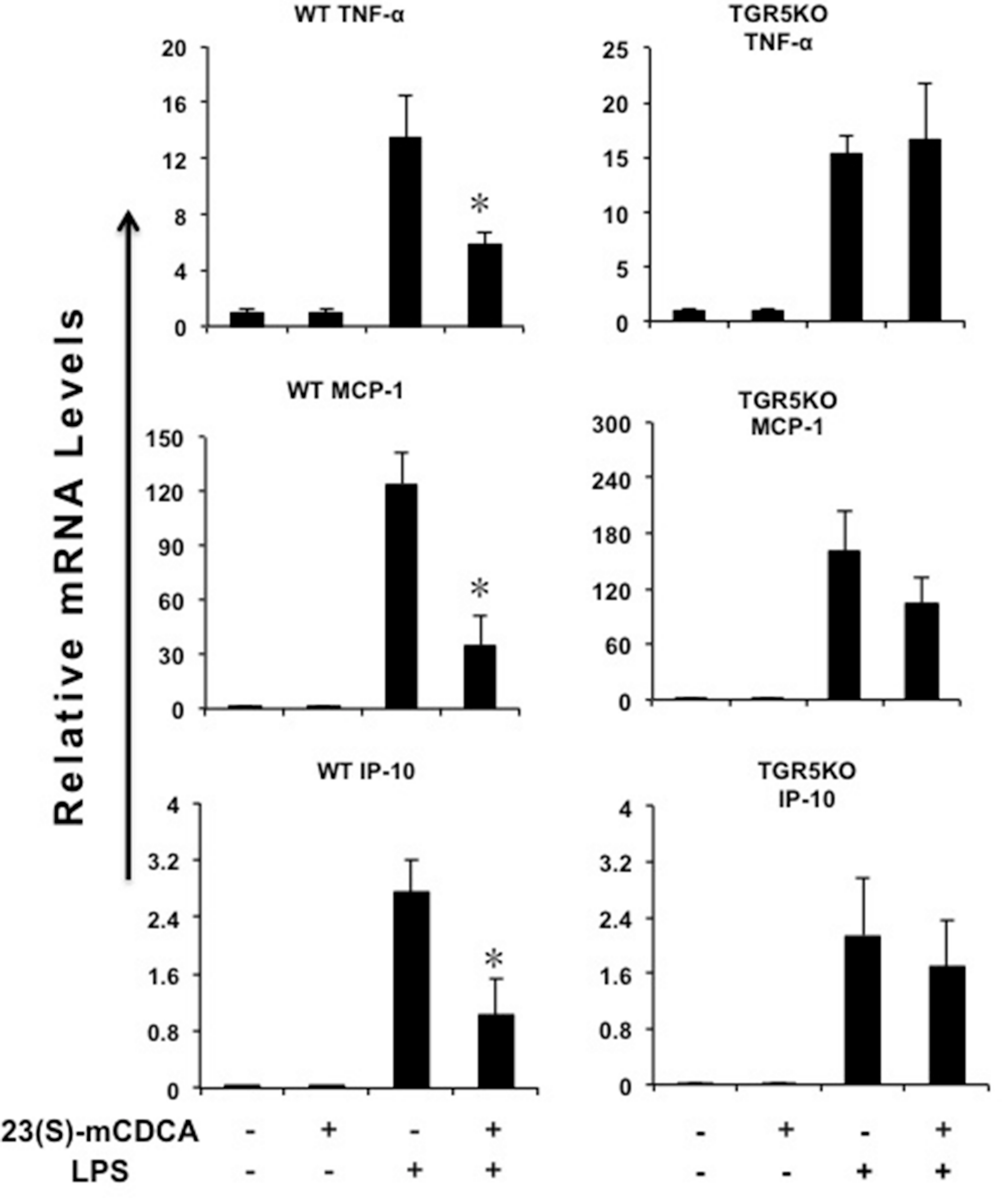
TGR5 activation suppresses kidney inflammation *in vivo* TGR5 ligand treatment repressed LPS-induced proinflammatory gene expression in WT, but not TGR5^−/−^ mouse kidney (*n* = 5–6). *P < 0.05 versus the only LPS-treated WT groups. Ligand, 23(S)-mCDCA.

23(S)m-CDCA-activated TGR5 suppressed LPS-induced TNF-α, monocyte chemoattractant protein-1 (MCP-1) and interferon-inducible protein 10 (IP-10) gene expression in WT kidney, but not TGR5^−/−^ kidney (Figure 1). In WT mouse kidney, LPS did not change the expression levels of TGR5 and 23(S)-mCDCA increased TGR5 expression level slightly but not significantly (Supplementary Figure 1).

### Activation of TGR5 reduced proliferation and migration of human kidney cancer cells

**Figure 2:**
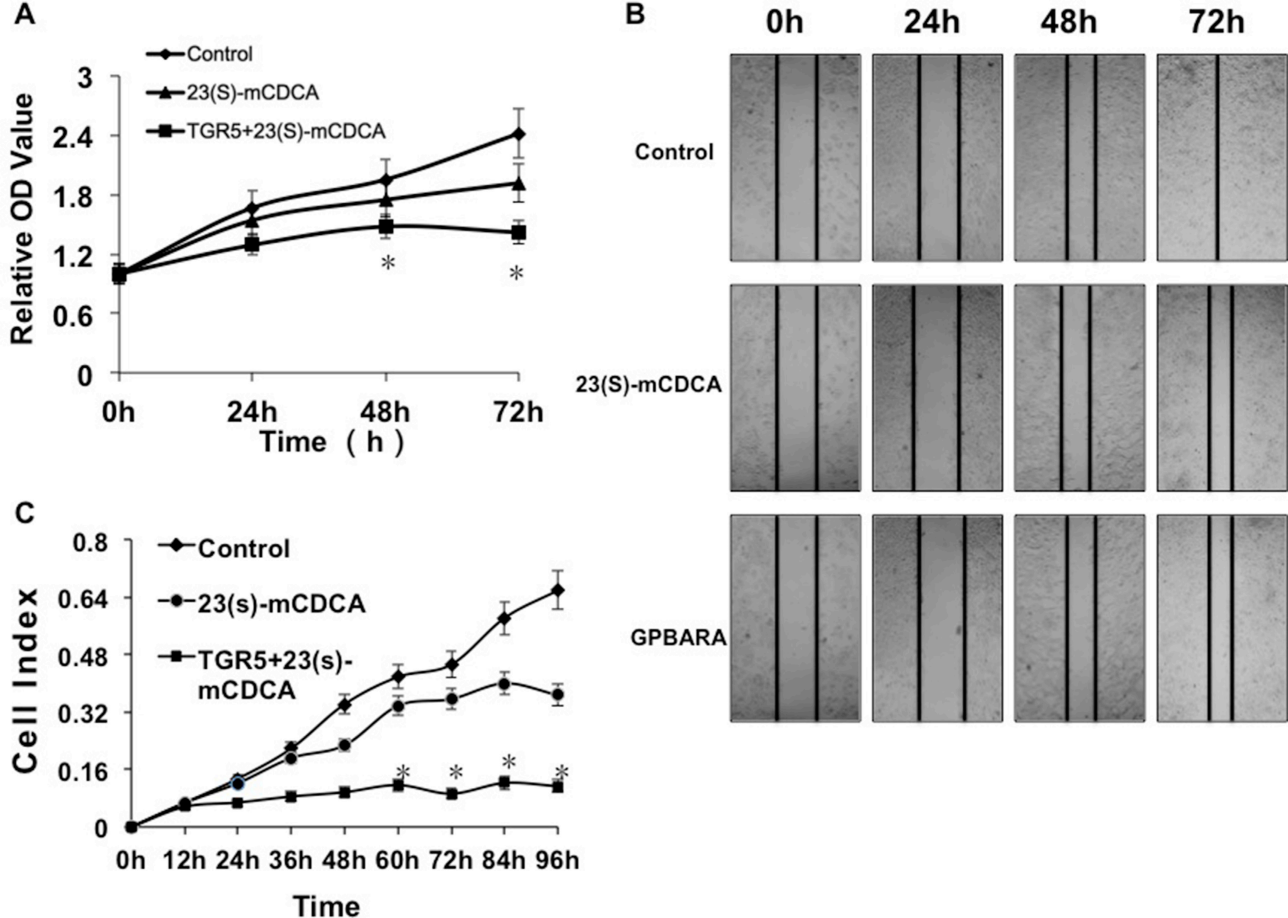
TGR5 activation impairs proliferation and migration of human kidney cancer cells (**A**) TGR5 activation by its ligand inhibited proliferation of HEK293 cells. Proliferation of cells was analyzed using MTT assay. TGR5 plasmid was transfected into HEK293 cells and then the ligand was added into the culture. After 24, 48 and 72 hours of treatment, MTT assay was performed to determine cell proliferation. *P < 0.05 versus the control groups (*n* = 3). (**B**) TGR5-transfected cells with ligand treatment exhibited a lower scratch closure rate than the controls in *in vitro* scratch assay (*n* = 3). The experiments were performed in triplicate and a representative of three independent experiments was shown. (**C**) *In vitro* cell migration assay shown that TGR5 activation inhibited HEK293 cell migration (*n* = 3). *P < 0.05 versus the control groups.

The potential of cells to proliferate, or to migrate is the most important cancer-causing factor. To determine how TGR5 affected kidney cancer cell growth and progression, we overexpressed TGR5 in HEK293 kidney cancer cells and determined whether activation of TGR5 by its ligands affected on cell proliferation and migration. As shown in MTT results, 23(S)-mCDCA treatment suppresses the growth of HEK293 cells slightly (Figure 2A). TGR5 overexpression enhanced this suppression (Figure 2A). TGR5 knockdown by TGR5-specific siRNA alleviated slightly the suppression (Supplementary Figure 2A). We also found that activation of TGR5 repressed the proliferation of renal carcinoma A498 cells (Supplementary Figure 3A). Meanwhile, in order to test human kidney cancer cell migration, *in vitro* scratch assay was done. Although TGR5 ligands did not affect wound closure of HEK293 cells (Supplementary Figure 4), the groups with activation of overexpressed-TGR5 by its ligands displayed a lower scratch closure rate than the control groups (Figure 2B). *In vitro* cell invasion assay was also performed using the xCELLigence^®^ RTCA DP instrument system. It was found that the groups with activation of overexpressed-TGR5 by its ligands exhibited lower migration compared with the controls (Figure 2C). TGR5 overexpression has been confirmed using Western blot assay (Supplementary Figure 5). 23(S)-mCDCA only suppressed cell migration (Figure 2C) but TGR5 knockdown by TGR5-specific siRNA alleviated the suppression at 36, 48 and 60 hours (Supplementary Figure 2B). These findings suggest that activation of TGR5 reduced human kidney cancer cell proliferation and migration, which may result in inhibiting kidney cancer development.

### TGR5 suppresses IκBα phosphorylation, p65 translocation and STAT3 phosphorylation

**Figure 3:**
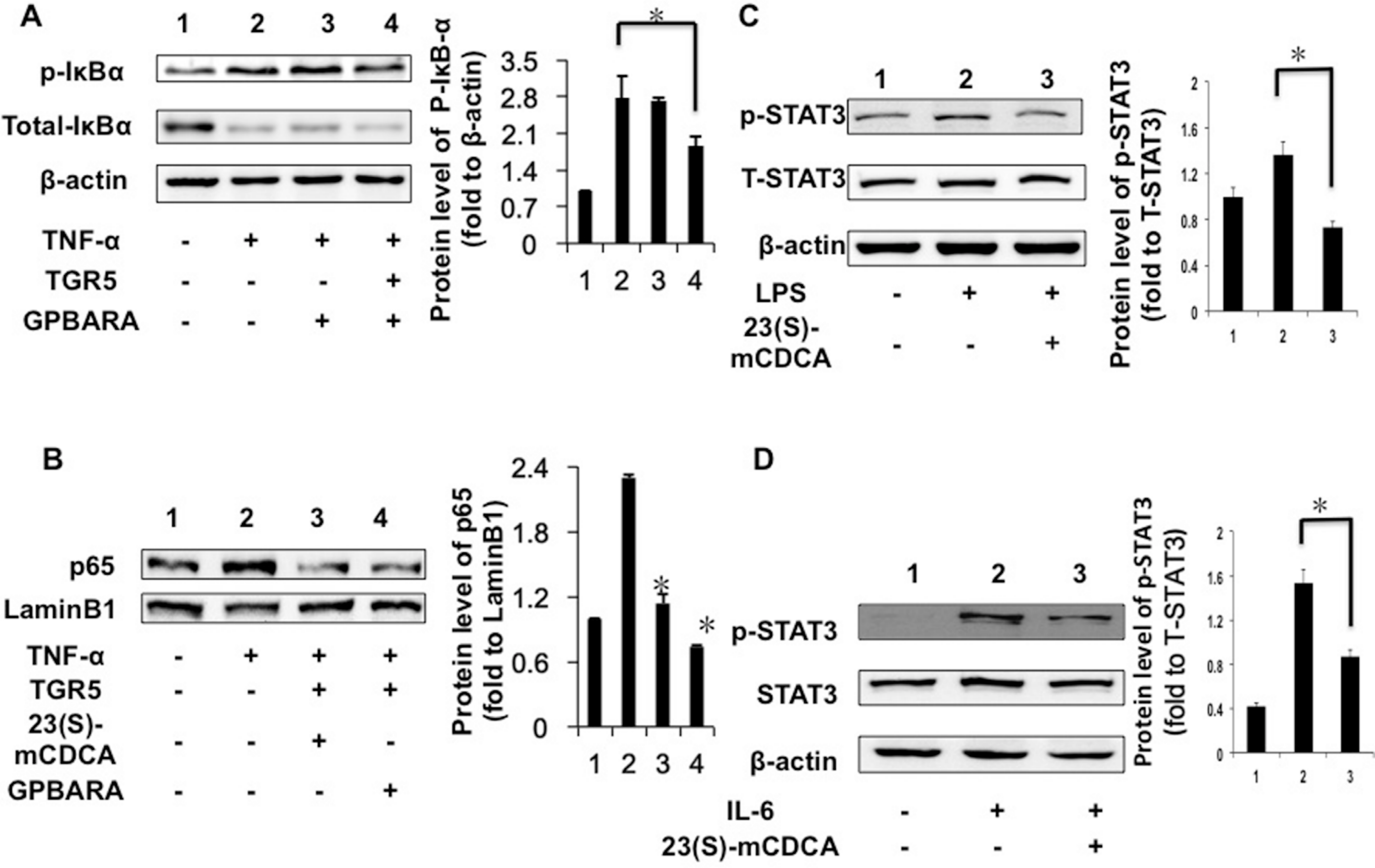
TGR5 inhibits IκBα phosphorylation, p65 translocation and STAT3 phosphorylation in kidney cancer cells (**A**) TGR5 overexpression with ligand treatment suppressed TNF-α-induced phosphorylation of IκBα in HEK293 cells. Cells were transfected with TGR5 plasmid and then treated with ligand for 24 hours. Finally, cells were treated with TNF-α (25 ng/mL) for 30 min. (*n* = 3) p-IκBα, phosphorylated IκBα. *P < 0.05. (**B**) TGR5 overexpression with ligand treatment suppressed TNF-α-induced the translocation of p65 in HEK293 cells. Cells were transfected with TGR5 plasmid and then were treated with the ligand GPBARA or 23(S)-mCDCA for 24 hours. Then cells were treated with TNF-α (25 ng/mL) for 30 min. *P < 0.05 versus the TNF-α-treated groups. (**C**) TGR5 ligand treatment suppressed LPS-induced phosphorylation of STAT3 in HEK293 cells. Cells were treated with ligand for 24 hours and then were treated with LPS (1 μg/mL) for 6 hours. (*n* = 3) p-STAT3, phosphorylated STAT3; T-STAT3, total STAT3. β-actin as a loading control. (**D**) TGR5 ligand treatment suppressed IL-6-induced p-STAT3 in HEK293 cells. Cells were treated with ligand for 24 hours and then were treated with IL-6 (12 ng/mL) for 4 hours. (*n* = 3) p-STAT3, phosphorylated STAT3 at Tyr705; T-STAT3, total STAT3. β-actin as a loading control. *P < 0.05.

Next, we found that TGR5 overexpression with ligand treatment (GPBARA) in HEK293 cells repressed TNF-α-stimulated IκBα phosphorylation by about 33% (Figure 3A). NF-κB activation can be stimulated via nuclear translocation of p65. TNF-α promoted the nuclear translocation of p65 in HEK293 cells (Figure 3B). TGR5 activation by its ligands suppressed p65 translocation promoted by TNF-α in HEK293 cells (Figure 3B).

STAT3 is considered as an important factor in inflammation and cancer development [12, 15, 21]. We found that, compared with the controls, LPS increased the levels of phosphorylated STAT3 (Figure 3C) in HEK293 cells. TGR5 activated by 23(S)-mCDCA treatment suppressed LPS-stimulated STAT3 phosphorylation by about 46% (Figure 3C). Furthermore, we used interleukin-6 (IL-6) induction to confirm this result. IL-6 increased the levels of phosphorylated STAT3 (Figure 3D). TGR5 activation by 23(S)-mCDCA suppresses IL-6-induced phosphorylation of STAT3 by about 43% (Figure 3D). It was also found that overexpressed-TGR5 activated by its ligand repressed STAT3 phosphorylation in renal carcinoma A498 cells (Supplementary Figure 3B).

### TGR5 activation suppresses gene expression mediated by NF-κB and STAT3 in kidney cancer cells

**Figure 4:**
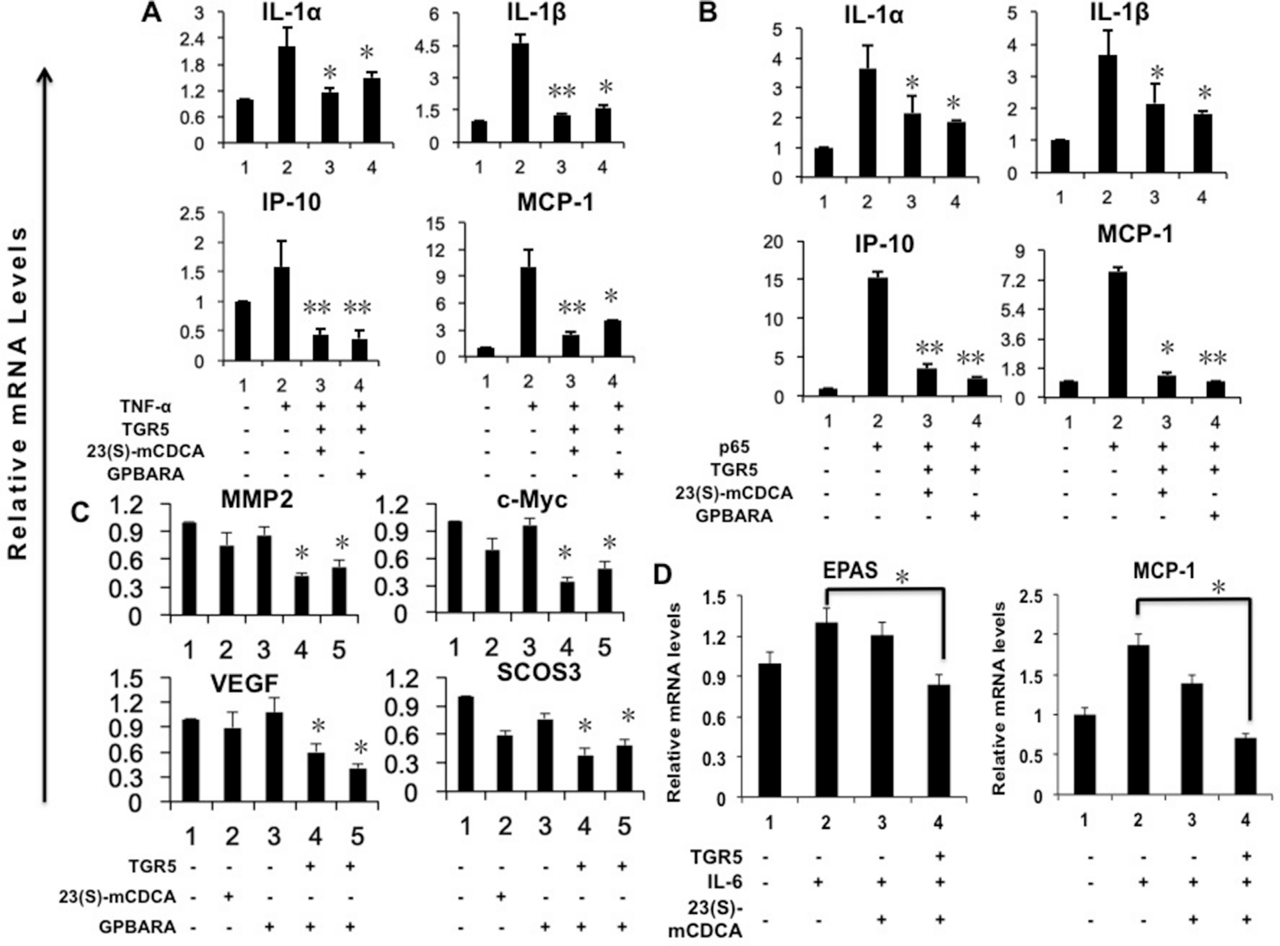
Activation of TGR5 antagonizes NF-κB and STAT3-mediated gene expression in kidney cancer cells (**A**) TGR5 overexpression with ligand treatment suppresses TNF-α-induced gene expression. HEK293 cells were transfected with the TGR5 expression plasmid or control plasmid. After transfection, cells were treated with GPBARA (3 μM), 23(S)-mCDCA (10 μM) or vehicle (DMSO) for 24 hours. Then cells were treated with TNF-α (15 ng/mL) for 1 hour. *P < 0.05, **P < 0.005 versus the TNF-α-treated group. (*n* = 3). (**B**) TGR5 overexpression with ligand treatment suppresses p65 overexpression-induced gene expression. HEK293 cells were transfected with the TGR5 and/or p65 expression plasmids or control plasmid. After transfection, cells were treated with GPBARA (3 μM), 23(S)-mCDCA (10 μM) or vehicle (DMSO) for 24 hours. *P < 0.05, **P < 0.005 versus the p65 overexpressed group. (*n* = 3). (**C**) TGR5 overexpression with ligand treatment suppresses STAT3-meidated gene expression. HEK293 cells were transfected with the TGR5 expression plasmid or control plasmid. After transfection, cells were treated with GPBARA (3 μM), 23(S)-mCDCA (10 μM) or vehicle (DMSO) for 24 hours. *P < 0.05 versus the control group (without any treatment). (*n* = 3). (**D**) TGR5 activation suppresses IL-6-induced gene expression. HEK293 cells were transfected with the TGR5 expression plasmid or control plasmid. After transfection, cells were treated with 23(S)-mCDCA (10 μM) or vehicle (DMSO) for 24 hours. Then cells were treated with IL-6 (20 ng/mL) for 6 hours. *P < 0.05 versus the LPS-treated group. (*n* = 3).

We previously reported that activation of TGR5 antagonizes gene expression mediated by NF-κB in hepatocytes and gastric cells [6, 20]. Here, we found that, in HEK293 kidney cancer cells, TGR5-transfected cells with the ligand treatment suppressed gene expression of IL-1α, IL-1β, IP-10, and MCP-1 induced by TNF-α or p65 overexpression (Figure 4A, 4B).

Next, we showed that in HEK293 kidney cells, overexpressed TGR5 induced by its ligands 23(S)-mCDCA or GPBARA suppressed gene expression of MMP2, c-Myc, VEGF and suppressor of cytokine signaling 3 (SOCS3) mediated by STAT3 (Figure 4C). Furthermore, overexpressed TGR5 activated by 23(S)-mCDCA suppressed gene expression of EPAS and MCP-1 induced by IL-6 (Figure 4D). *In vivo*, we used S3I-201, a inhibitor of STAT3, treated mice concurrently with TGR5 ligand INT-777. We found that concurrent treatment with INT-777 and S3I-201 suppressed LPS-induced the expression of proinflammatory genes IP-10 and MCP-1, which was similar that of the groups treated with INT-777 after LPS induction (Supplementary Figure 6). Concurrent treatment with INT-777 and S3I-201 enhanced the suppression of TNF-α expression mediated by INT-777 (Supplementary Figure 6).

We also tested whether TGR5 activation suppressed proinflammatory genes in renal carcinoma A498 cells. It was found that 23(S)-mCDCA suppressed IL-1β, IL-1α, MCP-1 and IP-10 gene expression and GPBARA suppressed MCP-1, IL-1α and IP-10 gene expression (Supplementary Figure 3C).

### TGR5 activation suppresses NF-κB transcriptional activity in kidney cells

**Figure 5:**
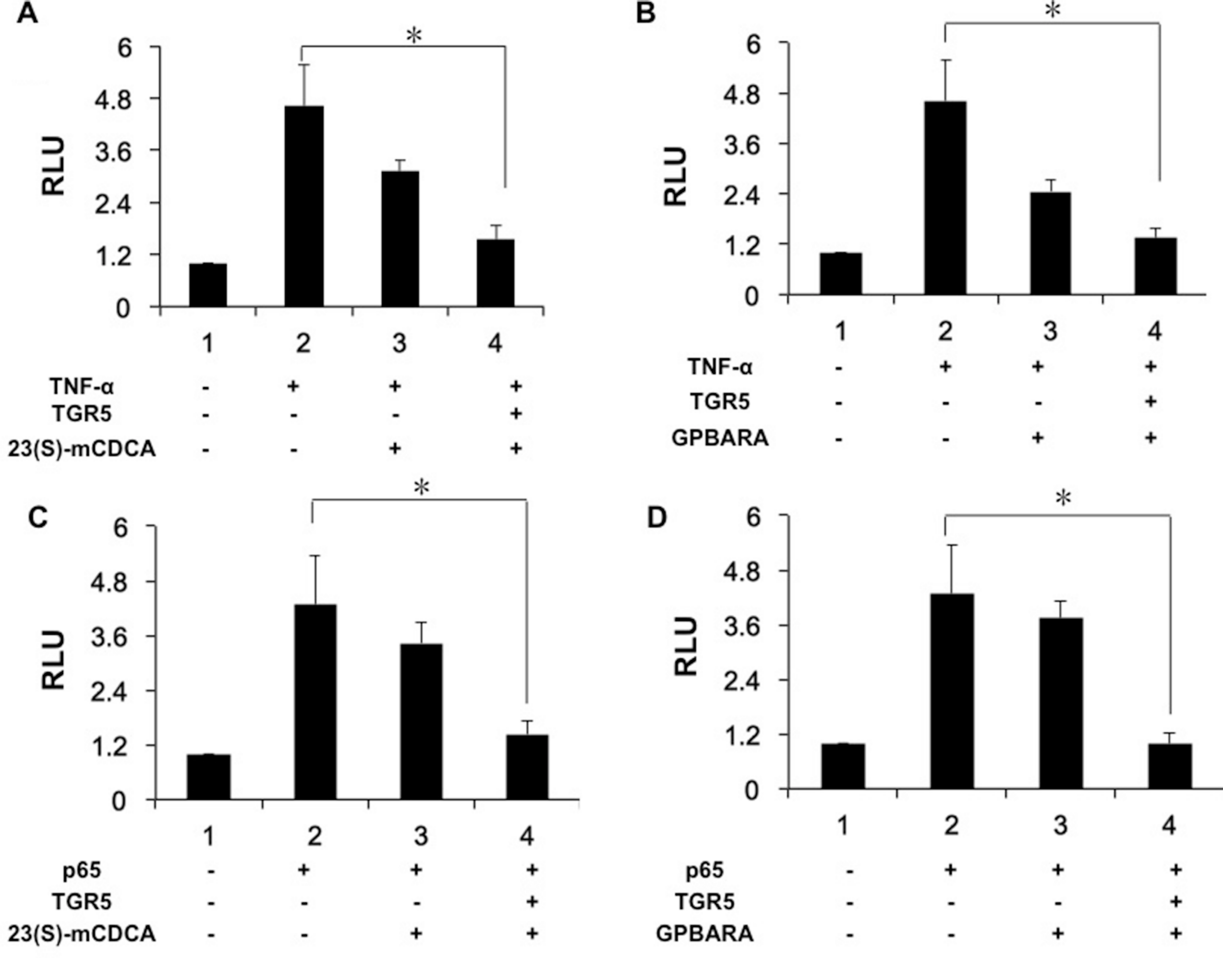
Activation of TGR5 antagonizes NF-κB transactivity (**A**, **B**) TGR5 suppressed NF-κB transactivity induced by TNF-α. HEK293 cells were cotransfected with the NF-κB reporter plasmid (pNF-κB-LUC), phRL-TK, and TGR5 expression plasmid. After transfection, cells were treated with 23(S)-mCDCA (10 μM) (A), GPBARA (3 μM) (B) or vehicle (DMSO) for 24 hours and then treated with TNF-α (15 ng/mL) for 6 hours. (**C**, **D**) TGR5 suppressed NF-κB transactivity induced by p65 overexpression. HEK293 cells were cotransfected with the NF-κB reporter plasmid (pNF-κB-LUC), phRL-TK, and TGR5 and p65 expression plasmids. After transfection, cells were treated with 23(S)-mCDCA (10μM) (C), GPBARA (3 μM) (D) or vehicle (DMSO) for 24 hours. *P < 0.05. RLU, relative luciferase units. (*n* = 3).

Next, we used TNF-α to increase NF-κB reporter activity (Figure 5A, 5B). TNF-α-induced NF-κB activation was repressed by both 23(S)-mCDCA and GPBARA treatment. Overexpression of TGR5 further enhanced this suppression (Figure 5A, 5B). Furthermore, we transfected p65 plasmid to the cells to activate the NF-κB reporter. P65 overexpression resulted in increasing NF-κB reporter activity by 4.3-fold (Figure 5C, 5D). NF-κB activity was inhibited by both TGR5 ligands with TGR5 overexpression (Figure 5C, 5D). We also found that TGR5 ligands suppressed p65 overexpression-induced NF-κB reporter activity in A498 cells (Supplementary Figure 3D).

## DISCUSSION

TGR5 not only regulates metabolic diseases and energy homeostasis but also participating in inflammation-associated diseases including cancer [11, 18, 20]. Few reports have been published about how TGR5 functions in kidney diseases. Our present work indicates that TGR5 may protect against kidney inflammation by regulating NF-κB. We demonstrate that TGR5 suppresses proliferation and migration of HEK293 kidney cancer cells. It is shown that TGR5 suppressed STAT3 pathway by antagonizing STAT3 phosphorylation and transcriptional activity in kidney cancer cells, thereby highlighting TGR5 as a therapeutic target for treatment of kidney diseases through antagonizing STAT3 signaling.

GPCRs not only regulate inflammatory response, but also mediate tumor progression. Some GPCRs promote the growth of tumor cells [23], while some GPCRs inhibit tumor development [24]. Currently, we noted that activation of TGR5 suppressed kidney cancer cell proliferation and migration possibly by suppressing both STAT3 and NF-κB pathways, which suggests that TGR5 is a potential kidney tumor suppressor. It should be mentioned that TGR5 has opposite biological effects in cancer cell proliferation. For example, Casaburi et al. and Hong et al. reported that TGR5 promoted human endometrial and human Barrett's cancer cell proliferation [25, 26], respectively, which are opposite results from ours. It suggests that the functions of TGR5 in cancer cell proliferation may be cell-type-dependent.

In summary, our results suggest that targeting TGR5 has a therapeutic potential for treatment of kidney inflammation or even kidney cancer. Activated TGR5 inhibits NF-κB and STAT3 signaling pathways, revealing that TGR5 ligands have potential as anti- inflammation and anticancer agents in kidney.

## MATERIALS AND METHODS

### Reagents and plasmids

LPS (from Escbricbia coli 0111:B4) and S3I-201 was purchased from Sigma Chemical (St Louis, MO). INT-777 was from MedChem Express. IL-6 was purchased from PeproTech. 23(S)-mCDCA was provided by Dr. Wendong Huang and Dr. Donna Yu (City of Hope, Duarte, CA). GPBARA (TGR5 Receptor Agonist, 3-(2-Chlorophenyl)-N-(4-chlorophenyl)-N,5-dimethylisoxazole-4-carboxamide) was purchased from BioVision (Milpitas, CA). The pmTGR5 expression vectors were created in our laboratory. The mouse TGR5 gene was cloned into pIRESneo3 (Clontech) plasmid to generate pmTGR5 overexpression plasmid. The p65 expression vector and the phRL-TK vector were kindly provided by Xufeng Chen and Akio Kruoda (both City of Hope, Duarte, CA), respectively. The NF-κB-dependent reporter (NF-κBx3-LUC) was provided by Dr. Peter Tontonoz (UCLA, Los Angeles, CA) and Dr. Bruce Blumberg (UCLA, Los Angeles, CA).

### Animals

Eight-week-old wild-typ (WT) (C57BL/6J) and TGR5^−/−^ female mice (on C57BL/6J background; Merck Research Laboratories, Kenilworth, NJ) were maintained in a pathogen-free animal facility under a standard 12-hour light-dark cycle. Mice were fed a diet containing 10 mg of 23(S)-mCDCA/kg diet or standard rodent chow for 3 days. After that, mice were fasted overnight and then injected intraperitoneally (i.p.) with a single dose of LPS (20 mg/kg) or phosphate-buffered saline (PBS), followed by feeding water ad libitum. Six hours after the injection, mice were killed by CO_2_ asphyxiation, and the kidney was removed for further analysis. The animal study proposal was approved by Beckman Research Institute of City of Hope Institutional Animal Care and Use Committee (IACUC). All animal experiments were carried out in accordance with an approved Beckman Research Institute of City of Hope Institutional Animal Care and Use Committee (IACUC) protocol.

### Cell culture and transfection

Kidney cancer cell line HEK293 was obtained from Institute of Basic Medical Sciences (IBMS) of Chinese Academy of Medical Sciences. Cells were grown in complete culture medium (RPMI-1640 [with L-glutamine] supplied with 10% (vol/vol) inactivated fetal calf serum and 1% (vol/vol) antibiotics-antimycotics). Cultures were fed with fresh medium twice weekly. For experiments, 6 × 10^5^ HEK293 cells were seeded in 60 mm culture dishes with complete culture medium. Transient transfection of HEK293 cells with TGR5 expression plasmid or the empty plasmid (without TGR5 cDNA, as a control) was performed using Lipofectamine 2000 (Invitrogen, Carlsbad, CA). Twenty-four hours after transfection, cells were pre-treated with 23(S)-mCDCA (10 μM) or GPBARA (3 μM) for one day. Then cells were treated with or without TNF-α (15 ng/mL) for one hour or IL-6 (20 ng/mL) for 6 hours. Then cells were harvested for Quantitative Real-Time PCR analysis. For protein assay, cells were pre-treated with 23(S)-mCDCA (10 μM) or GPBARA (3 μM) for one day. Then cells were treated with TNF-α, LPS or IL-6 for the indicated times. Finally, cells were collected for total protein isolation and Western blot assay. For luciferase assay, transient transfection of HEK293 cells with the NF-κB reporter plasmid, phRL-TK, and/or TGR5 expression plasmid was performed using Lipofectamine 2000 (Invitrogen, Carlsbad, CA). Twenty-four hours after transfection, cells were pre-treated with 23(S)-mCDCA (10 μM), GPBARA (3 μM) or vehicle (dimethyl sulfoxide (DMSO)) for 24 hours. Then cells were treated with/without TNF-α (15 ng/mL). After 6 hours of incubation, cells were harvested and the luciferase activity was determined using a dual-luciferase reporter assay system in accordance with the manufacturer's instructions (Promega, Madison, WI). Luciferase activities were normalized by co-transfection of the control thymidine kinase-driven Renilla luciferase plasmid, phRL-TK. Data are expressed as relative fold activation to that of non-stimulated (−) sets.

### RNA isolation and quantitative real-time polymerase chain reaction

Total RNA was extracted from HEK293 cells using Tri-Reagent (Molecular Research Center, Inc., Cincinnati, OH). Quantitative real-time PCR was performed using the Power SYBR Green PCR Master Mix protocol (Applied Biosystems, Foster City, CA). Amplification of β-actin was used as an internal reference. β-Actin primers were obtained from Ambion, Inc. (Austin, TX). Quantitative PCR analysis was conducted using the ABI 7300 Sequence Detection System. Primers sequences are available on request.

### Immunoblot analysis

At indicated time points after treatment, HEK293 cells were lysed for 30 minutes with lysis buffer and centrifuged at 12,000 × g at 4°C for 15 minutes. The samples were resolved by 10% sodium dodecyl sulfate-polyacrylamide gel electrophoresis, transferred to nitrocellulose membranes, and blotted using primary antibodies (Cell Signaling Technology). The membranes were washed with Tris Buffered Saline with 0.1% Tween^®^ 20 (TBST) and then incubated with anti-rabbit secondary antibody conjugated to horseradish peroxidase (HRP) (1:5000) (Thermo Scientific, Waltham, MA). Bands on blots were visualized using Tanon 5200 enhanced chemiluminescence (ECL) detection system (Tanon, China) and quantified with a computerized digital imaging system using Tanon software.

### Cell proliferation assay

Cell proliferation was measured using the MTT assay every 24 hours. Briefly, 100 μL of cell suspension (5 × 10^4^/mL) was added to each well of a 96-well plate and incubated at 37°C for 24 hours. Then TGR5 plasmid was transfected to cells. After 24 hours, cells were treated with 10 μM of 23(S)-mCDCA. After 24, 48, or 72 hours of treatment, MTT reagent was added into cells. After 4 hours of incubation, 150 μL of dimethyl sulfoxide was added to dissolve formazan crystals, and optical density was measured at 570 nm.

### *In vitro* scratch assay

For detection of cell migration by *in vitro* scratch assay, HEK293 cells were cultured to confluent monolayers and then were treated with 23(S)-mCDCA (10 μM), GPBARA (3 μM) or vehicle (DMSO) for 24 hours, and then wounded by removing a 300–500 μm-wide strip of cells across the well with a standard 200 μL pipette tip. Wounded monolayers were washed twice to remove non-adherent cells. After indicated times of incubation, wound healing was recorded under a light microscopy.

### Cell migration assay

The xCELLigence^®^ RTCA DP instrument system (ACEA Biosciences, San Diego, CA) was applied to monitor cell migration by using CIM-plates. HEK293 cells were seeded in normal culture medium for 24 h. Then cells were transfected with TGR5 plasmid or control plasmid. Before cells were transferred, for CIM-plates, normal culture medium was then placed in the lower chamber. The plate was left to settle for 30 min at room temperature (RT) in sterile conditions. The upper chamber was then mounted and 50 μl of serum free medium was added to each well and left to equilibrate in the incubator for 1 h at 37°C and 5% CO2. After the incubation, a background reading was taken for each well. Then transfected cells were transferred into the upper chamber in serum medium with ligands. Cell index were measured with the RTCA software at 9 scans at 12 hour intervals until the end of the experiment (up to 96 h).

### Statistics

All data represent at least three independent experiments and are expressed as the mean ± SD. The two-way analysis of variance (ANOVA), followed by Bonferroni's post-hoc test, was performed. A *P* value less than 0.05 was considered significant.

## SUPPLEMENTARY MATERIALS FIGURES


