# Supplementary material for: The G-protein-coupled bile acid receptor Gpbar1 (TGR5) protects against renal inflammation and renal cancer cell proliferation and migration through antagonizing NF-κB and STAT3 signaling pathways
Source: Oncotarget. 2017 Apr 29;8(33):54378–87. doi: 10.18632/oncotarget.17533 (PMC5589588; doi:10.18632/oncotarget.17533)
Supplement: Supplementary file 1 [file oncotarget-08-54378-s001.pdf]

The G-protein-coupled bile acid receptor Gpbar1 (TGR5) protects against renal inflammation and renal cancer cell proliferation and migration through antagonizing NF-κB and STAT3 signaling pathways

Supplementary Materials

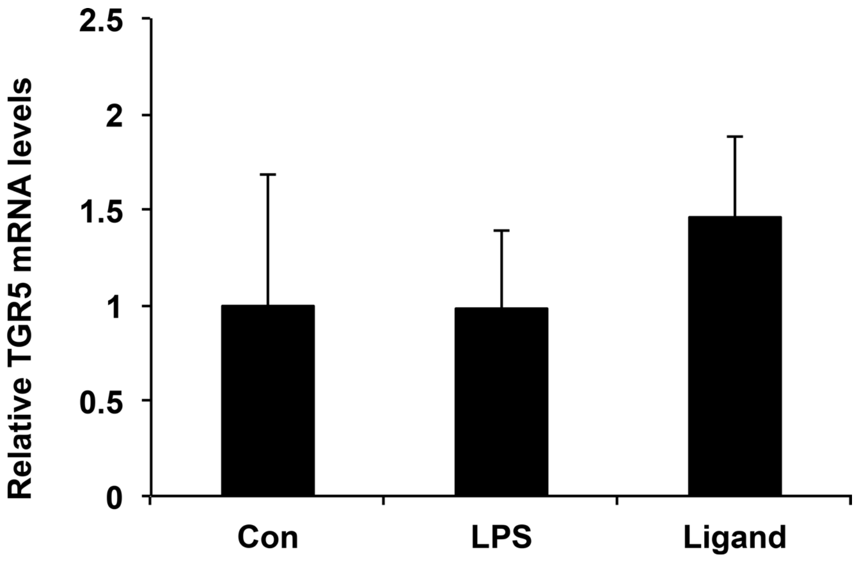

Supplementary Figure 1: The effects of LPS or ligand on TGR5 mRNA levels in mouse kidney. Eight-week-old wild-type (WT) (C57BL/6J) female mice were fed a diet containing 10 mg of 23(S)-mCDCA/kg diet or standard rodent chow for 3 days. After that, mice were fasted overnight and then injected intraperitoneally (i.p.) with a single dose of LPS (20 mg/kg) or phosphate-buffered saline (PBS), followed by feeding water ad libitum. Six hours after the injection, mice were killed by CO_2 asphyxiation, and the kidney was removed for RNA isolation and QRT-PCR. *n* = 5–6.

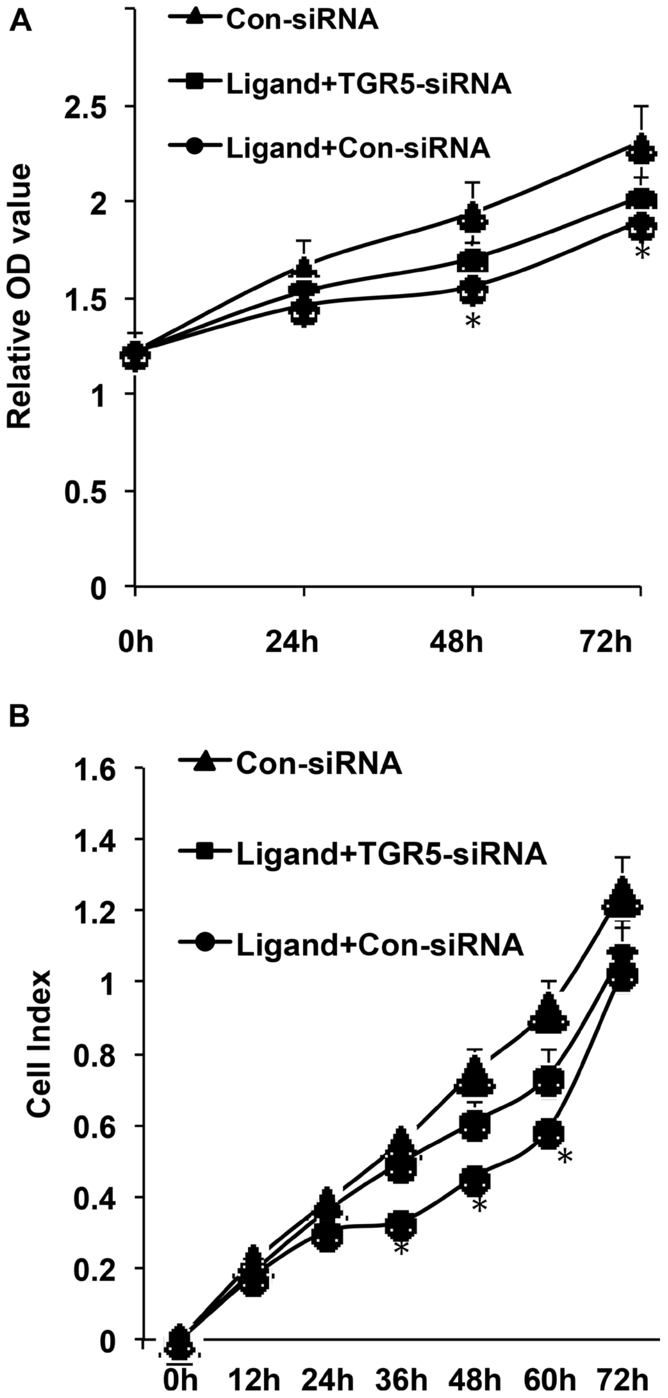

Supplementary Figure 2: The effects of TGR5 knockdown by anti-TGR5 siRNA on suppression of HEK293 cell proliferation and migration mediated by TGR5 ligand (23(S)-mCDCA). (**A**) Proliferation of cells was analyzed using MTT assay. Control siRNA or TGR5 siRNA (Ribobio, Guangzhou, P. R. China) was transfected into HEK293 cells and then the ligand was added into the culture. After 24, 48 and 72 hours of treatment, MTT assay was performed to determine cell proliferation. ($n = 3$). (**B**) HEK293 cells were seeded in normal culture medium for 24 h. Then cells were transfected with control siRNA or TGR5 siRNA. Before cells were transferred, for CIM-plates, normal culture medium was then placed in the lower chamber. The plate was left to settle for 30 min at room temperature (RT) in sterile conditions. The upper chamber was then mounted and 50 μl of serum free medium was added to each well and left to equilibrate in the incubator for 1 h at 37°C and 5% CO2. After the incubation, a background reading was taken for each well. Then transfected cells were transferred into the upper chamber in serum medium with ligands. Cell index were measured with the RTCA software at 9 scans at 12 hour intervals until the end of the experiment (up to 72 h). $*P < 0.05$ versus the control groups.

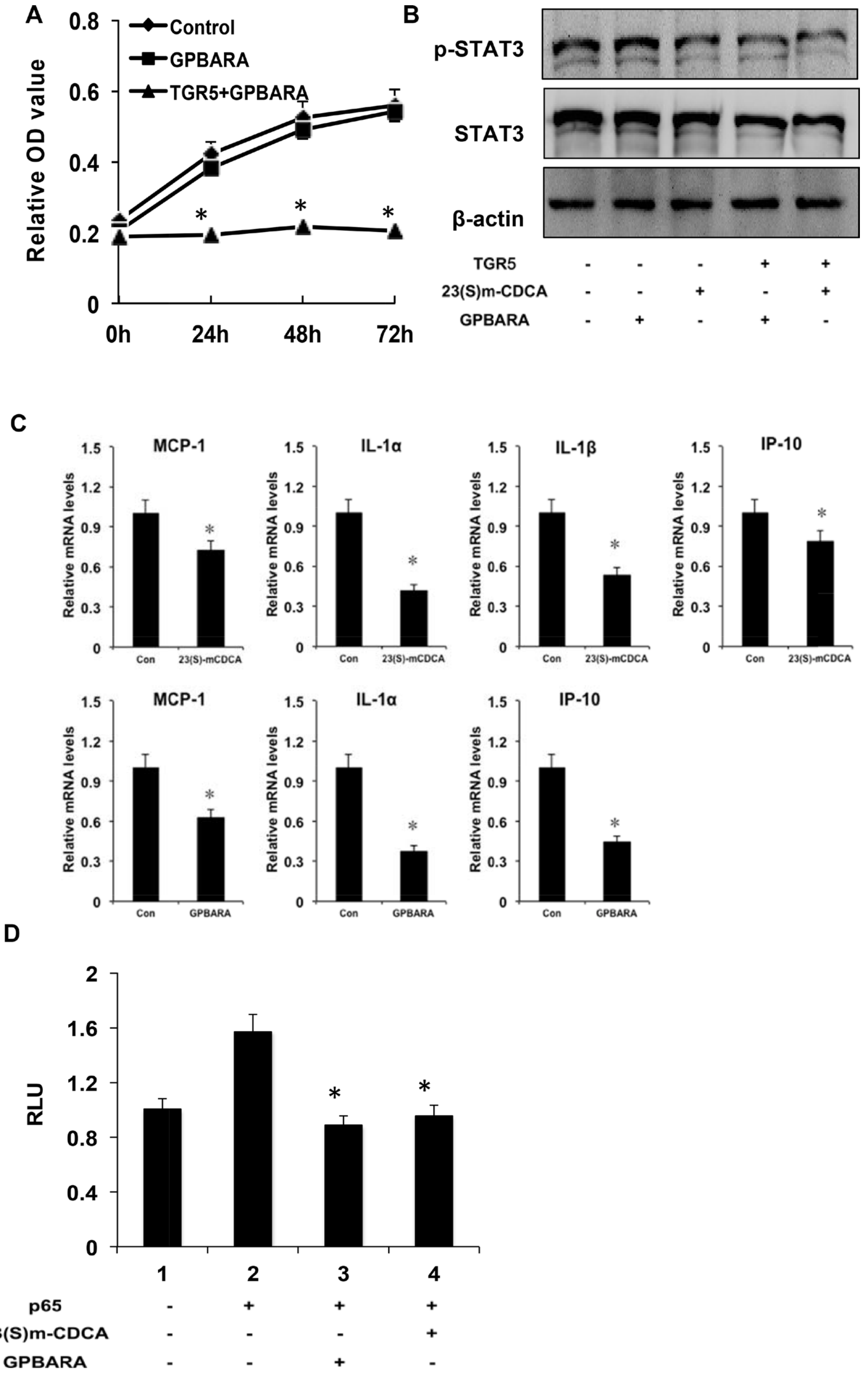

Supplementary Figure 3: The effects of TGR5 activation on A498 renal carcinoma cells. (**A**) Proliferation of cells was suppressed by TGR5 activation. Proliferation of cells was analyzed using MTT assay. TGR5 plasmid or control plasmid was transfected into A498 cells and then the ligand GPBARA (5 μM) was added into the culture. After 24, 48 and 72 hours of treatment, MTT assay was performed to determine cell proliferation. ($n = 3$) *$P < 0.05$ versus the control groups. (**B**) TGR5 overexpression with ligand treatment suppressed the phosphorylation of STAT3 in A498 cells. Cells were transfected with TGR5 plasmid or control plasmid and then treated with ligands (10 μM of 23(S)-mCDCA or 5 μM of GPBARA) for 24 hours. p-STAT3, phosphorylated STAT3. ($n = 3$) (**C**) TGR5 ligand treatment for 24 hours suppressed inflammatory gene expression in A498 cells. 23(S)-mCDCA, 10 μM; GPBARA, 5 μM. (**D**) TGR5 suppressed NF-κB transactivity induced by p65 overexpression in A498 cells. A498 cells were cotransfected with the NF-κB reporter plasmid (pNF-κB-LUC), phRL-TK, and TGR5 and p65 expression plasmids. After transfection, cells were treated with 23(S)-mCDCA (10 μM), GPBARA (5 μM) or vehicle (DMSO) for 24 hours. *$P < 0.05$. RLU, relative luciferase units. ($n = 3$).

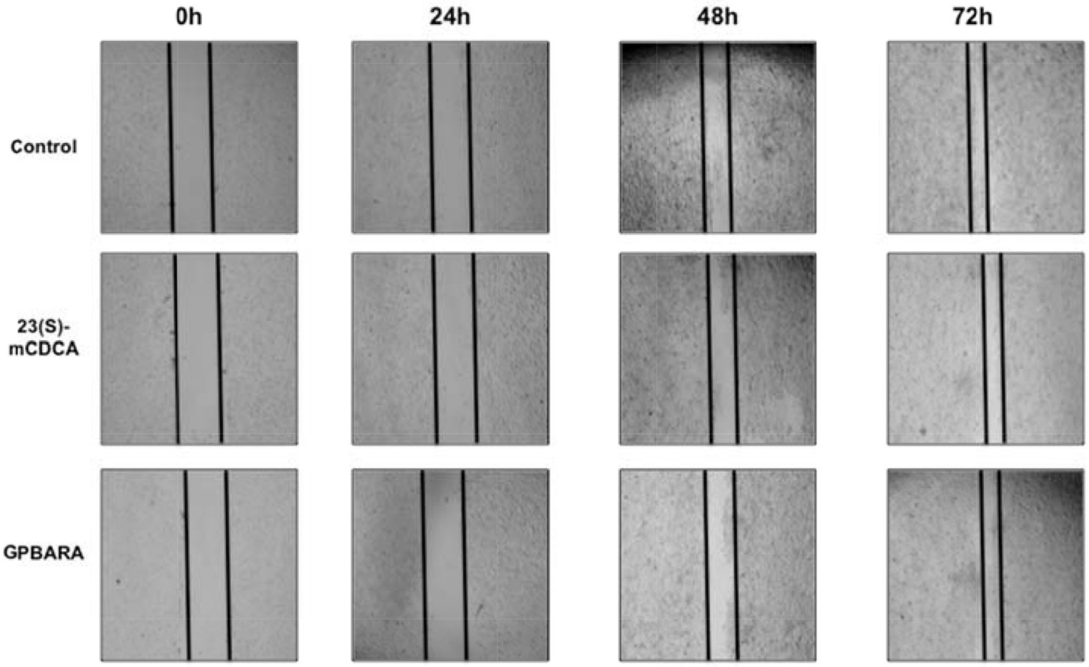

Supplementary Figure 4: Only TGR5 ligand treatment did not change wound closure in HEK293 cells. HEK293 cells were treated with GPBARA (3 μM) or 23(S)-mCDCA (10 μM). The experiments were performed in triplicate and a representative of three independent experiments was shown.

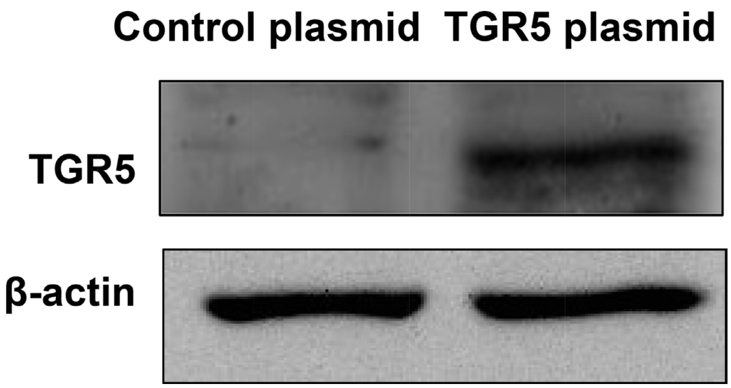

Supplementary Figure 5: TGR5 overexpression was confirmed by Western blot. Control plasmid or TGR5 plasmid was transfected into HEK293 cells. Total proteins including membrane proteins were isolated for Western blot analysis. β-actin as an inernal control. Anti-TGR5 antibody was from Abcam (Rockville, MD, USA).

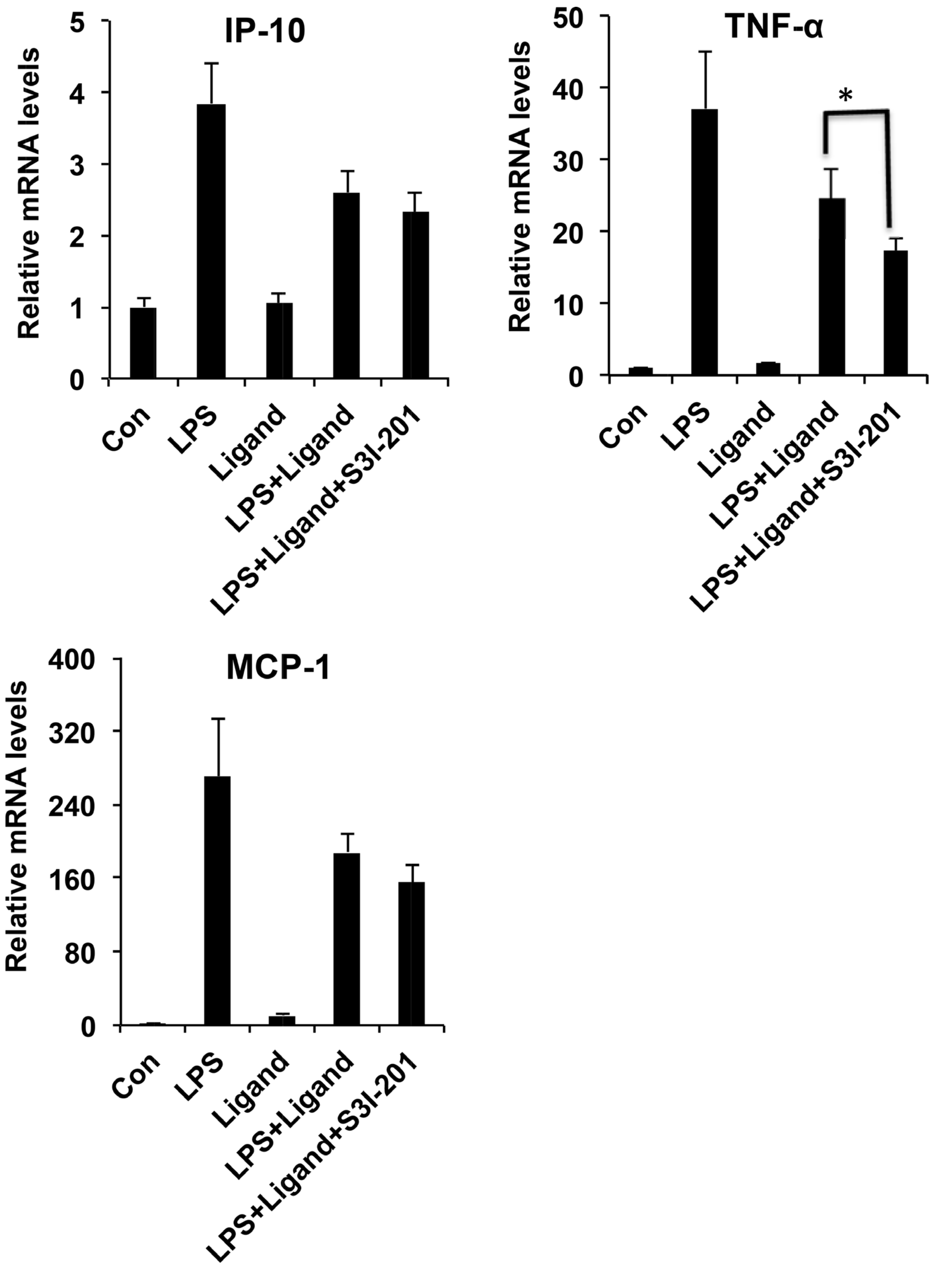

Supplementary Figure 6: The mRNA levels of proinflammatory genes in mouse kidney concurrently treated with of STAT3 inhibitor, S3I-201 and TGR5 ligand. Eight-week-old wild-typ (WT) (C57BL/6J) female mice Mice were fed a diet containing 30 mg of INT-777/kg diet or standard rodent chow and cocurrently injected intraperitoneally (i.p.) with S3I-201 (5 mg/Kg body weight) per day for 3 days. After that, mice were fasted overnight and then injected (i.p.) with a single dose of LPS (20 mg/kg) or phosphate-buffered saline (PBS), followed by feeding water ad libitum. Six hours after the injection, mice were killed and the kidney was removed for QRT-PCR analysis. **P* < 0.05. (*n* = 4–5).
